# A Biomarker Panel of Radiation-Upregulated miRNA as Signature for Ionizing Radiation Exposure

**DOI:** 10.3390/life10120361

**Published:** 2020-12-18

**Authors:** Man Song, Dafei Xie, Shanshan Gao, Chen-Jun Bai, Mao-Xiang Zhu, Hua Guan, Ping-Kun Zhou

**Affiliations:** Beijing Key Laboratory for Radiobiology, Beijing Institute of Radiation Medicine, Beijing 100850, China; songman002@163.com (M.S.); xiedafei@sina.com (D.X.); gaoshanbprc@163.com (S.G.); bccjcc1990@aliyun.com (C.-J.B.)

**Keywords:** miRNA, biomarkers, ionizing radiation, miRNA-gene network, gene expression

## Abstract

Ionizing radiation causes serious injury to the human body and has long-time impacts on health. It is important to find optimal biomarkers for the early quick screening of exposed individuals. A series of miRNAs signatures have been developed as the new biomarkers for diagnosis, survival, and prognostic prediction of cancers. Here, we have identified the ionizing radiation-inducible miRNAs profile through microarray analysis. The biological functions were predicted for the top six upregulated miRNAs by 4 Gy γ-rays: miR-1246, miR-1307-3p, miR-3197, miR-4267, miR-5096 and miR-7641. The miRNA-gene network and target gene-pathway network analyses revealed that *DNAH3* is the target gene associated with all the six miRNAs. *GOLGB1* is related to 4 miRNAs and other 26 genes targeted by 3 miRNAs. The upregulation of fifteen miRNAs were further verified at 4 h and 24 h after 0 to 10 Gy irradiation in the human lymphoblastoid AHH-1 cells, and some demonstrated a dose-dependent increased. Six miRNAs, including miR-145, miR-663, miR-1273g-3p, miR-6090, miR-6727-5p and miR-7641, were validated to be dose-dependently upregulated at 4 h or 24 h post-irradiation in both AHH-1 and human peripheral blood lymphocytes irradiated ex vivo. This six-miRNA signature displays the superiority as a radiation biomarker for the translational application of screening and assessment of radiation exposed individuals.

## 1. Introduction

In the events of nuclear disasters, nuclear radiation accidents as well as space radiation hazardous, the tissue damage caused by ionizing radiation can be catastrophic, resulting in acute and long-term health detriments and even death [1,2,3,4,5]. Development of robust quick and high-throughput biomarkers based on an individual’s early biological response is crucial for accurate assessment of the level of exposure and sickness severity, and the eventual medical decisions. Radiosensitive biomarkers not only indicate weather you are exposed to radiation or not, and the exposure level, but also provide more information about radiation-induced tissues damage and any changes in biological processes. Traditional biomarkers based on DNA damage, such as micronuclei formation, chromosomal aberration and γ-H2AX foci formation [6,7,8,9,10], have been developed for many years. However, these methods are time consuming and require complex analysis by skilled workers, amongst other considerable limitations. Therefore, high throughput, quick response, simple and efficient biomarkers urgently need to be developed to overcome the limitations or replace the traditional biomarkers.

In the recent years, many research projects have been dedicated to the discovery of radiation and other environmental factors exposure biomarkers in blood samples or serum, such as many microRNAs [11,12,13,14,15,16] and proteins [17,18,19,20,21]. MicroRNA (miRNA) is non-coding small RNA comprised of approximately 20 bases and are a kind of classic negative regulator of gene expression. they are involved in regulating gene expression via the post-transcriptional regulation of messenger RNA and the inhibition of protein translation [22]. MiRNA expression has identified signatures associated with diagnosis, progression, prognosis and response to treatment in different kinds of cancers [23,24]. Although there have been many studies on the expression and function of miRNAs in irradiated cell, more panels of miRNAs signatures, especially those with radiation dose-dependency for the assessment of radiation exposure need to be further developed and improved.

In this study, the differentially expressed miRNAs were screened by miRNA microarray and profiled in human umbilical vascular endothelial cells (HUVEC) after γ-ray irradiation. The miRNA-gene network and target gene-pathway network of top six of the upregulated expressed miRNAs have been depicted through bioinformatical analysis. In order to explore the differentially expressed miRNAs, especially those with the radiation dose-dependent expression changes, for the translational unitality of radiation exposure biomarkers, the human lymphoblast cell line AHH-1 and peripheral blood were used to further sort out the miRNA biomarkers of radiation exposure from the radiation-upregulated miRNAs identified by microarray analysis. 15 upregulated miRNAs by γ-ray irradiation have been confirmed by qRT-PCR, and a numbers of miRNAs molecules were further verified to be dose-dependently upregulated from 0 to 10 Gy. Our current study provided the valuable information on radiation-inducible miRNAs for translational application of biomarkers to distinguish and assess population of radiation exposure.

## 2. Materials and Methods

### 2.1. Cell Culture

The suspended human lymphoblast AHH-1 cells and the human umbilical vein endothelial (HUVEC) cells were cultured in RPMI-1640 containing 10% fetal bovine serum (FBS), 100 U/mL of penicillin and 100 µg/mL of gentamycin in a humidified incubator at 37 °C under 5% CO_2_ atmosphere. At one day before irradiation, the AHH-1 cells were sub-cultured in 25-cm culture flask, and the HUVEC cells were sub-cultured on 100 mm petri dishes. The cells were irradiated with ^60^Co γ-rays at a dose rate of 0.5771 Gy/min at the room temperature.

### 2.2. Human Peripheral Blood Samples

The peripheral blood samples from three healthy adult volunteers (males) were collected and subjected to quantification of the miRNA expression levels. The subjects had no history of chronic disease, substance abuse, smoking, or toxic chemical exposure. In addition, they had not been exposed to radiation and had no history of viral infections during the six months preceding the study. The peripheral blood samples (20 mL) were collected from each subject via venipuncture into Vacutainers (BD Biosciences, Franklin Lakes, NJ, USA). A total of six different radiation doses were applied (0, 2, 4, 6, 8 and 10 Gy). The blood samples were divided into six plastic centrifuge tubes (1 tube/dose group), and irradiated with a single dose of ^60^Co γ-rays at a dosage rate of 0.5771 Gy/min. Following irradiation, the blood samples were removed to 25-cm culture flask and incubated at 37 °C for 4 h and 24 h in equal volume of RPMI-1640 medium supplemented with 10% FBS. Lymphocytes RNA purification from the human blood samples was performed by using QIAamp RNA Blood Mini Kit (Catalog No. 52304, QIAGEN, Hilden, Germany) in accordance with the manufacturer’s protocol.

### 2.3. miRNA Extraction and μParaflo™ MicroRNA Microarray Assay

Following exposure to different doses of ^60^Co γ-rays, the cells were collected after 4 h and 24 h of irradiation. The total RNA was extracted from the irradiated HUVEC cells and control cells using TRlzol reagent (Invitrogen), as recommended by the manufacturer. We examined the quality of RNA by RIN-value and 1% gel electrophoresis, OD260/OD280 value is between 1.8–2.0, and 1% Gel electrophoresis is used to estimate RNA degraded or not. The miRNA microarray analyses were performed in the LC Bio Co. LTD (Hangzhou, China) based on the following kits and microarray hybridization procedures according to the manufacturers’ instructions: LC Sciences microRNA Microarray-Single (miRBase 21.0) was used. Hybridization was performed overnight on a μParaflo microfluidic chip using a micro-circulation pump (Atactic Technologies). On the microfluidic chip, each detection probe consisted of a chemically modified nucleotide coding segment complementary to target microRNA (from miRBase, http://www.mirbase.org/) and a spacer segment of polyethylene glycol to extend the coding segment away from the substrate. The detection probes were prepared by in situ synthesis using PGR (photogenerated reagent) chemistry. The hybridization melting temperatures were balanced by chemical modifications of the detection probes. After RNA hybridization, tag-conjugating Cy3 dye were circulated through the microfluidic chip for dye staining. Fluorescence images were collected using a laser scanner (GenePix 4000B, Molecular Device, Santa Clara Valley, CA, USA) and digitized using Array-Pro image analysis software (Media Cybernetics, Silver Spzing, MD, USA). Data were analyzed by first subtracting the background and then normalizing the signals using a LOWESS filter (Locally-weighted Regression). Differentially expressed miRNAs were identified and analyzed. *p*-value and false discovery rate (FDR) were calculated to evaluate the significance of differential expression of miRNAs before and after irradiation. For a fold-change >3, a threshold of 0.01 was chosen for the *p*-values.

### 2.4. Real-Time Quantitative PCR (RT–qPCR)

Total RNA (1–3 µg) was used to reverse-transcribe cDNA. The Roche TaqMan microRNA expression assay was used to quantitate the mature miRNA expression following the manufacturer’s protocol. U6 was used as an internal control for miRNA expression. PCR primers were used in this study are listed in Table S1. RT-qPCR was performed with a Bio-Rad iCycler & iQ Real-time PCR systems (Bio-Rad) and the Fluorescence-labeled FAM Hairpin-it microRNA and U6 snRNA Normalization TR-PCR Quantitation Kit (Catalog No. E22001–E22010, Gene Pharma, Shanghai, China). Each sample was tested thrice, and the miRNA expression in the untreated control was set as to generate the relative expression level in the treated cells. miRNAs primer sequences used in this study are listed in Supplementary data.

### 2.5. Bioinformatical Analyses

#### 2.5.1. Prediction of Target Genes

An active project in on-line database DIANA TOOLS named microT-CDS (http://www.microrna.gr/microT-CDS.) was used for prediction of target genes of significantly differentially expressed miRNAs. MicroT-CDS is the fifth version of the microT algorithm with an increased sensitivity. It is among the most prevalent tools dedicated to miRNA target prediction/functional analyses (Details on DIANA-microT web server v5.0: service integration into miRNA functional analysis workflows, Functional microRNA targets in protein coding sequences).

#### 2.5.2. Gene Ontology (GO) Analysis

GO is a consortium-based dataset providing information on functions of genes. It is a comprehensive and computational model for large-scale molecular biology and systems biology analyses in biomedical research. To investigate the molecular functions of genes and their products, GO covers three domains: cellular components (CC), molecular functions (MF) and biological processes (BP). Fisher’s exact test was used to determine whether a significant difference existed between the target genes and other genes. A *p*-value threshold of 0.05 was used.

#### 2.5.3. Enrichment Analyses of Pathways

We conducted pathway enrichment analysis of target genes of the identified human significantly differentially expressed miRNAs by the Database for Annotation, Visualization, and Integrated Discovery (DAVID) tool based on KEGG and identified enriched clusters of target genes with *p* ≤ 0.05. DAVID bioinformatics resources provide an integrated biological database and a repository of analytic tools for systematicexploration of biological meaning of gene sets DAVID. The default parameters in the tool were used and enriched pathways were ranked according to their enrichment scores.

#### 2.5.4. Construction of the miRNA-Gene Network and Target Gene-Pathway Network

After a thorough integration and manual curation, 3888 interactions among seven human significantly differentially expressed miRNAs and 3465 predicted target genes were obtained. The miRNA-gene network was constructed accordingly. A link was placed between a miRNA and a gene when the gene was predicted to be targeted by the miRNA by the DIANA TOOLS microT-CDS. Circles in red and blue correspond to miRNAs and their predicted target genes, respectively.

The predicted target genes related to >1 significantly differentially expressed miRNA, and their enriched pathways were used as nodes of the target gene-pathway network. A link was placed between the gene and the pathway if it was enriched in the pathway. Nodes in blue and red correspond to genes and their associated pathways, respectively. The miRNA-gene and target gene-pathway networks were visualized and illustrated using Cytoscape software.

### 2.6. Statistical Analysis

All statistical tests were performed by using the statistical package for the IBM-SPSS Statistics Ver.21.0. (IBM Corp., Armonk, NY, USA). Variables with normal distribution were expressed as mean ± SD. For quantitative variables, the Student’s *t*-test was used for the comparison between the two groups, and the Bonferroni’s post-hoc test was used to evaluate multiple groups, with *p* < 0.05 considered to indicate statistical significance of the results.

## 3. Results

### 3.1. Identification of Radiation-Induced miRNAs and Target Gene Prediction in HUVEC Cells

In order to obtain radiation-induced miRNAs, we collected two batches of HUVEC cells for miRNA microarray analysis after 4 Gy γ-ray irradiation. *p* > 0.01 and the detection signals values < 500 were removed. In the first batch of experiment, 117 and 98 miRNAs were identified at 0.5 h and 2 h after 4 Gy irradiation, respectively. In the second batch of experiment, 10 and 28 miRNAs were identified at 0.5 h and 2 h after 4 Gy irradiation, respectively. We compared the two batches and found that much more miRNAs were detected in Batch 1, for which clustering analysis was conducted. The heatmaps of changed miRNAs at 0.5 h and 2 h after 4 Gy of γ-ray irradiation were shown in Figures S1 and S2, respectively, the blue color indicates down-regulated miRNAs, and red color indicates up-regulated miRNA. The fold change values and the *p* values of miRNA detection signal are shown in Tables S2 and S3, respectively.

We considered three-times fold change as the threshold, where four miRNAs significantly changed both at 0.5 h and 2 h, respectively, in Batch 1, and 4 and 6 miRNAs significantly changed at 0.5 h and 2 h, respectively, in Batch 2. The top six miRNAs were miR-1246, miR-1307-3p, miR-3197, miR-4267, miR-5096 and miR-7641. The details of expression changes as detected by microarrays of these miRNAs were listed in Table 1. The target genes of the top 6 miRNAs are listed in Figure S3, a total of 3465 genes were analyzed as candidate target genes. Further analysis revealed that has-miR-4267 and has-miR-5096 could regulate >1000 genes. Both the miRNAs were significantly upregulated at 0.5 h after irradiation in both the batches. The hsa-miR-7641 and hsa-miR-1307-3p regulated 170 and 22 target genes, respectively. In addition, 3069 (88.6%) genes were regulated by only one miRNA, 368 (10.6%) genes are regulated by two miRNAs, and 26 (0.8%) genes were regulated by three miRNAs. *DNAH3* and *GOLGB1* were regulated by six and four miRNAs, respectively. *DNAH3* is a type of dynein involved in producing force for ciliary beating by using energy from ATP hydrolysis, and *GOLGB1* is a widely expressed large coiled-coil protein and Golgi integral membrane protein associated with neuropathies.

### 3.2. Annotation and Enrichment Pathways of Predicted miRNAs Target Genes

**Figure 1:**
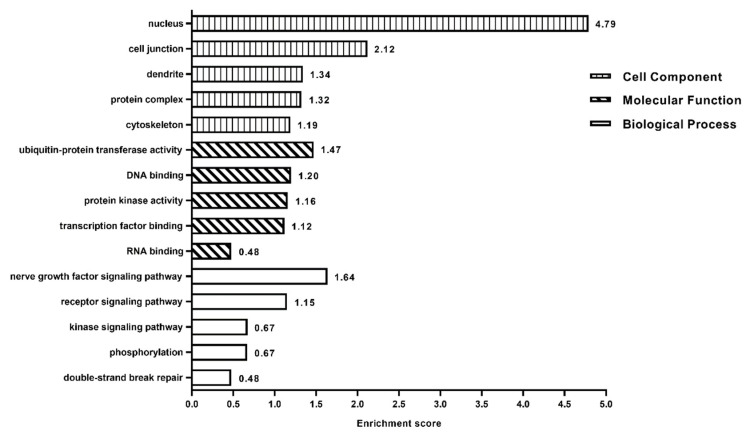
Clusters of miRNAs target genes for biological process (BP), cellular component (CC), and molecular function (MF) based on Gene Ontology (GO).

The enrichment analyses of biological process (BP), cellular component (CC) and molecular function (MF) for the 396 target genes based on Gene Ontology (GO) by DAVID Bioinformatics Resources 6.8 (https://david.ncifcrf.gov/home.jsp) (Figure 1). GO analyses shown that the clusters ranked 1–3 in BP are the nerve growth factor signaling pathway, receptor signaling pathway and kinase signaling pathway, respectively. The clusters that ranked 1–3 in CC include nucleus, cell junction and dendrite. The top three clusters in MF included ubiquitin-protein transferase activity, DNA binding, and protein kinase activity. The enrichment pathways of miRNAs target genes were conducted with reference to the Kyoto Encyclopedia of Genes and Genomes (KEGG). A total of 191 target genes were simultaneously regulated by has-miR-1307-3p and has-miR-7641, which were enriched in pathways of focal adhesion, rap1 signaling pathway, and regulation of actin cytoskeleton. There were total of 396 target genes related to 29 pathways in Table S4, including 13 signal transduction-related pathways, such as phosphatidylinositol signaling system, cGMP-PKG signaling pathway, oxytocin signaling pathway, and five cancer-related pathways, such as for glioma and non-small cell lung cancer (Figure 1).

### 3.3. miRNA-Gene Network and Target Gene-Pathway Network

**Figure 2:**
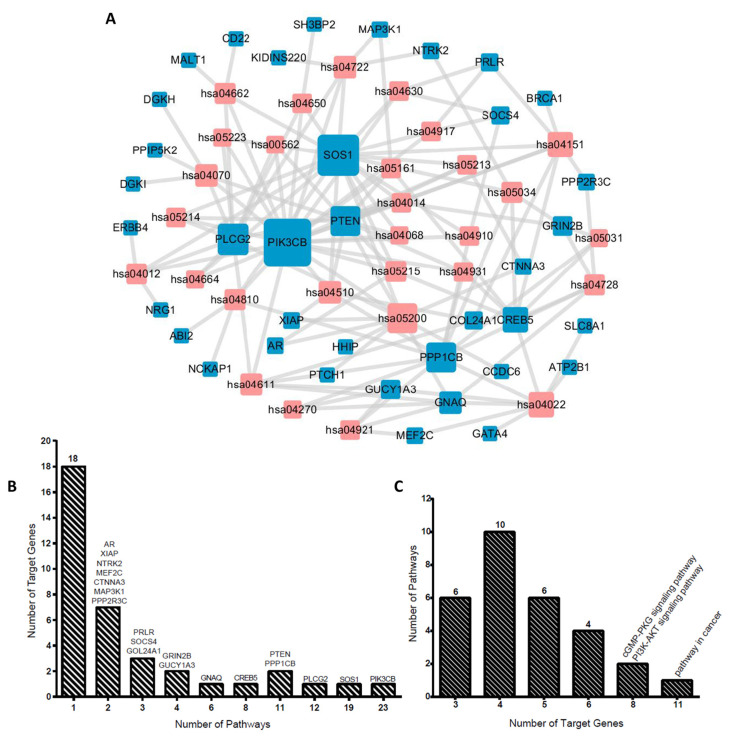
Gene-signaling pathway network of top 6 miRNAs target genes in HUVEC cells after irradiation. (**A**) The conjunction between a gene and the signaling pathway when the gene was predicted to be involved in the pathway with reference to Kyoto Encyclopedia of Genes and Genomes (KEGG). Nodes in red refer to the pathways, while those in blue refer to the target genes. (**B**) Distribution of numbers of target genes with numbers of signaling pathways in the network. (**C**) Distribution of numbers of signaling pathways with numbers of target genes in the network.

We explored interrelation among the top six miRNAs and their target genes, target genes and signaling pathways. A total of 3465 target genes of top six miRNAs were involved in 3688 gene interactions and the miRNA-gene network in Table S5. Considering that the number of genes were >1000, which is not very clear as per the PDF versions, and that the scalable pictures were viewed under the Cytoscape software. Some miRNAs target genes >1000, such as hsa-miR-4267 and hsa-miR-5096, and one gene was regulated by multiple miRNAs, for instance, *DNAH3* was regulated by six miRNAs, *GOLGB1* was regulated by four miRNAs, and other 26 genes were regulated by three miRNAs. The target gene-pathway network was established based on 396 target genes and 29 pathways, and there were a total 139 pairs of target gene-pathway connections, with each gene connected to approximately four pathways on an average (Figure 2A). As shown in Figure 2B, 28 (75.68%) miRNA target genes were involved in fewer signaling pathways. For instance, 18 genes were only related to one signaling pathway, which indicated the specific regulating relationships. *ABI2* was only related to the regulation of actin cytoskeleton, while *BRCA1* was only involved in the PI3K-Akt signaling pathway. Meanwhile, six (16.22%) miRNA target genes were involved in multiple signaling pathways, for example, *GNAQ* gene was involved in the regulation of six signal pathways and *CREB5* gene was related to the regulation of eight signal pathways. We further evaluated signaling pathways involving miRNA target genes (Figure 2C), as some signaling pathway involved very few genes, such as six signaling pathways were only involved in three miRNAs target genes, while 10 signaling pathways were only involved in four miRNAs target genes.

### 3.4. Confirmation of Differentially Expressed miRNAs Signature in Human Lymphoblast Cells AHH-1

**Figure 3:**
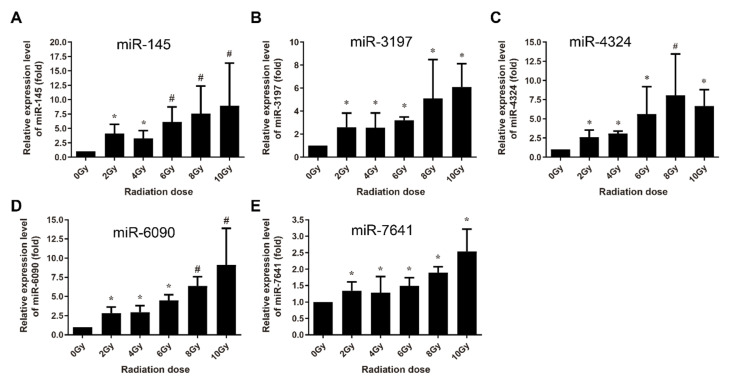
qRT-PCR verification of radiation dose-dependently upregulation of miRNAs in AHH-1 cell at 4 h after 0–10 Gy of γ-rays irradiation. (**A**–**E**): Respectively representation of the dose response-expression changes of miR-145, miR-3197, miR-4324, miR-6090, and miR-7641 in AHH-1 at 4 h after exposure to the ionizing radiation. Data represent the mean ± standard deviation from 3 independent experiments. As compared with 0 Gy group, * *p* < 0.05, # *p* < 0.01.

**Figure 4:**
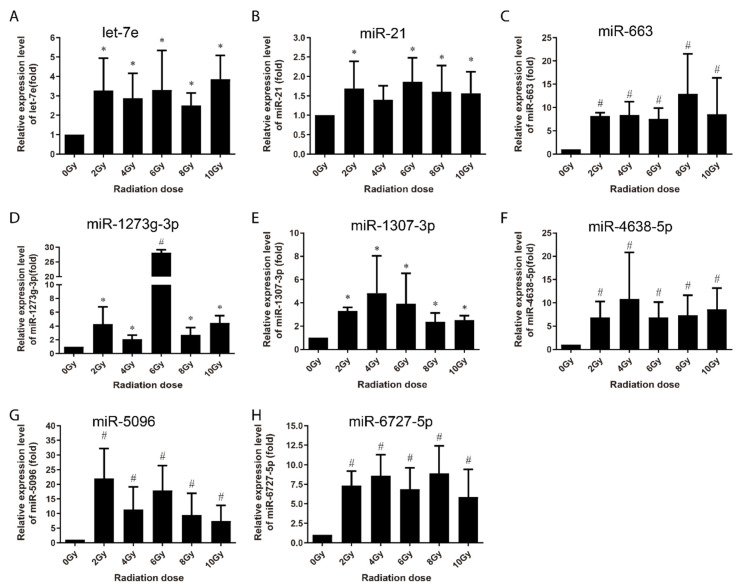
qRT-PCR verification of the upregulated expression of miRNAs in AHH-1 cell at 4 h after exposure to certain doses (or a dose range) of γ-rays irradiation. (**A**–**H**): Respectively representation of the expression changes of let-7e, miR-21, miR-663, miR-1273g-3p, miR-1307-3p, miR-4638-5p, miR-5096, and miR-6727-5p in AHH-1 cells at 4 h after irradiation. Data represent the mean ± standard deviation from 3 independent experiments. As compared with 0 Gy group, * *p* < 0.05, # *p* < 0.01.

In order to explore the translational utility of miRNAs for the biomarkers or biodosimetry of radiation exposure, the normally growing human lymphoblastoid AHH-1 cells were used to systematically verify the signature of a group of differentially expressed miRNAs induced via ionizing radiation by qRT-PCR. The AHH-1 cells cultures were irradiated from 0 Gy to 10 Gy dose using cobalt γ-ray source, and the cells were harvested 4 h and 24 h after irradiation, and the small RNA fraction was recovered from the total RNA. For each sample, the expression values were normalized to the U6 snRNA, and we calculated the expression levels relative to the 0 Gy control group. At 4 h post-irradiation, 5 miRNAs (miR-145, miR-3197, miR-4324, miR-6090 and miR-7641) were identified to be significantly upregulated in a dose-dependent manner from 0 to 8 Gy or up to 10 Gy (Figure 3), while another 8 miRNAs were found to be upregulated by radiation, but without obvious dose-dependency (Figure 4).

**Figure 5:**
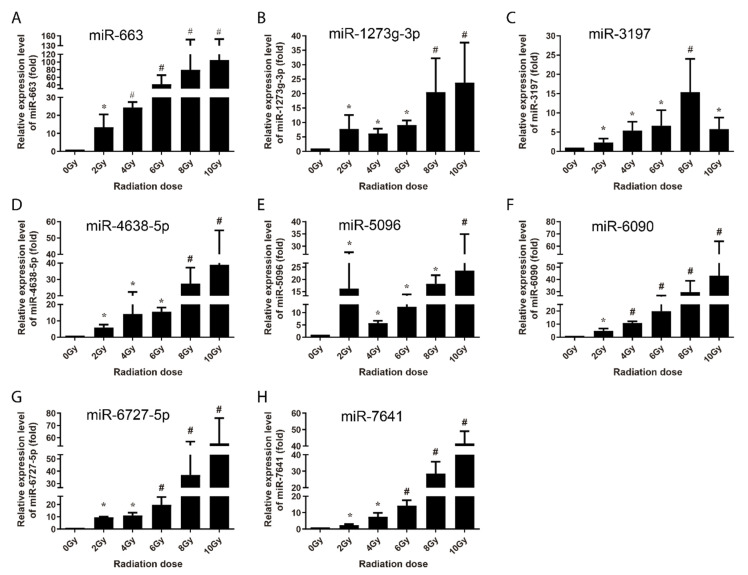
qRT-PCR verification of radiation dose-dependent upregulation of miRNAs in AHH-1 cell at 24 h after 0–10 Gy of exposure to γ-rays irradiation. (**A**–**H**): Respectively representation of the dose response-expression changes of miR-663, miR-1273g-3p, miR-3197, miR-4638-5p, miR-5096, miR-6090, miR-6727-5p, and miR-7641 in AHH-1 cells at 24 h after irradiation. Data represent the mean ± standard deviation from 3 independent experiments. As compared with 0 Gy group, * *p* < 0.05, # *p* < 0.01.

**Figure 6:**
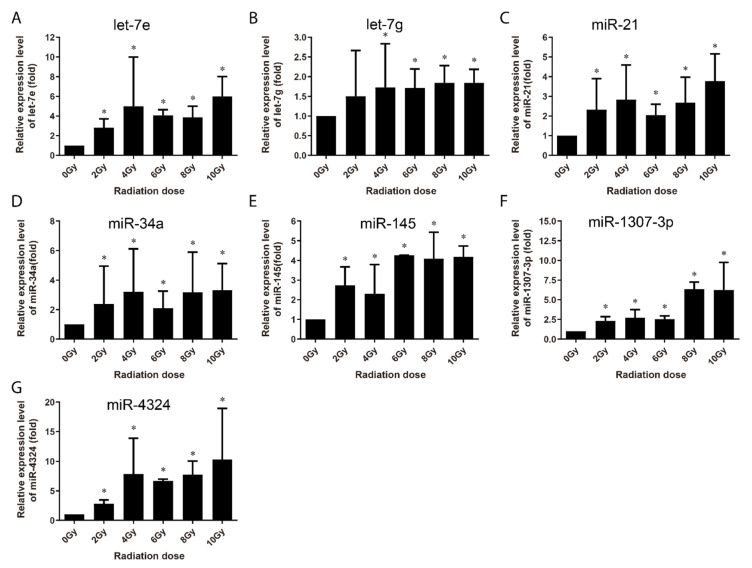
qRT-PCR verification of the upregulated expression of miRNAs in AHH-1 cell at 24 h after exposure to certain doses (or a dose range) of γ-rays irradiation. (**A**–**G**): Respectively representation of the expression changes of let-7e, let-7g, miR-21, miR-34a, miR-145, miR-1307-3p, and miR-4324 in AHH-1 cells at 24 h after irradiation. Data represent expressed as the mean ± standard deviation of the mean of 3 independent experiments. As compared with 0 Gy group, * *p* < 0.05.

At 24 h post-irradiation, 15 miRNAs were identified to have been upregulated (Figure 5 and Figure 6). Among them, eight miRNAs (miR-663, miR-1273g-3p, miR-3197, miR-4638-5p, miR-5090, miR-6090, miR-6727-5p and miR-7641) showed increased expression in a dose-dependent manner from 0 to 8 Gy or up to 10 Gy (Figure 5), while the other seven were upregulated at certain dose or dose or in a certain dose range (Figure 6). It is thus clear that 13 of these 15 miRNAs (except let-7g and miR-34a) were upregulated at both 4 h and 24 h post-irradiation and three miRNAs (i.e., miR-3197, miR-6090 and miR-7641) were dose-dependently upregulated at both the time points. These 15 miRNAs were further subjected to validation in human peripheral blood samples.

### 3.5. Validation of Radiation-Upregulated miRNAs in Human Peripheral Blood Cells as Radiation Exposure Biomarkers

**Figure 7:**
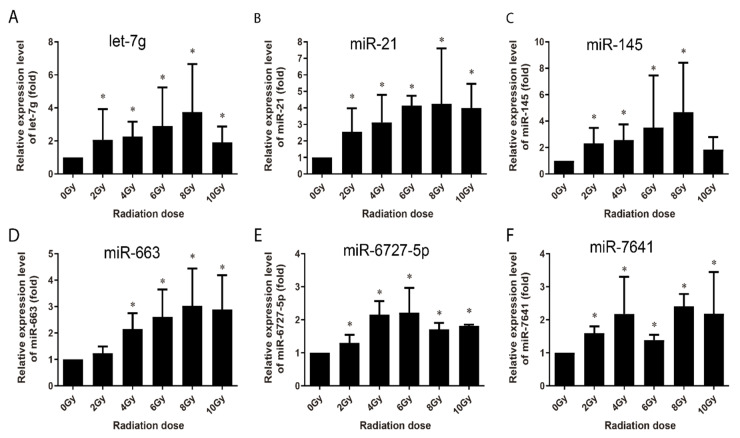
qRT-PCR validation of the upregulated expression of miRNAs in human peripheral blood lymphocytes collected from 3 healthy adult donors at 4 h after irradiation with different dosed ex vivo. (**A**–**F**) Respectively representation of the expression changes of let-7g, miR-21, miR-145, miR-663, miR-6727-5p, and miR-7641 in HPBL cells at 4 h after irradiation ex-vivo. Data represent the mean ± standard deviation from 3 volunteers’ blood samples. Three irradiation experiments were performed and detected for each sample. As compared with 0 Gy group, * *p* < 0.05.

**Figure 8:**
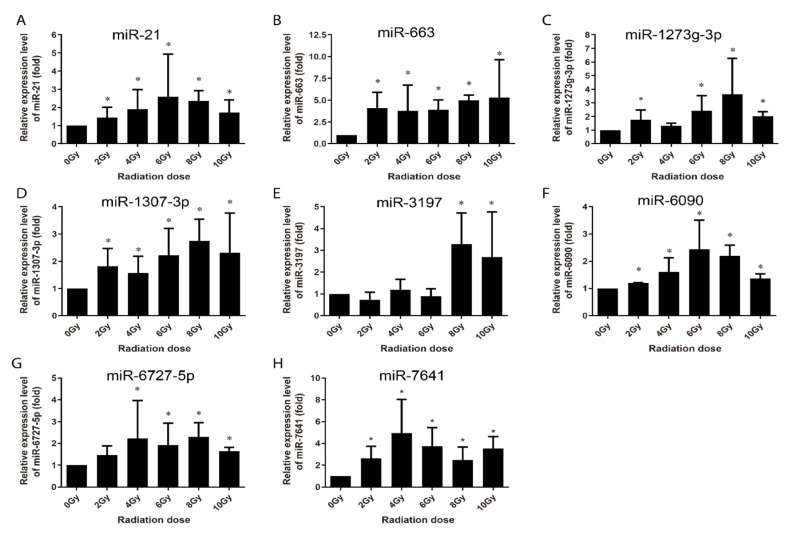
qRT-PCR validation of the upregulated expression of miRNAs in human peripheral blood lymphocytes collected from 3 healthy adult donors at 24 h after different dose of irradiation ex vivo. (**A**–**H**) Respectively representation of the expression changes of miR-21, miR-663, miR-1273g-3p, miR-1307-3p, miR-3179, miR-6090, miR-6727-5p, and miR-7641 in HPBL cells at 24 h after irradiation ex-vivo. Data represent the mean ± standard deviation from 3 volunteers’ blood samples. Three irradiation experiments were performed and detected for each sample. As compared with 0 Gy group, * *p* < 0.05.

The alterations in the radiation-induced miRNA were investigated in human peripheral blood samples collected from three healthy voluntary donors. The samples were analyzed 4 h and 24 h following ex vivo irradiation with 0, 2, 4, 6, 8 and 10 Gy of γ-rays. Human peripheral blood lymphocytes (HPBLs) were isolated, and miRNAs copy numbers were assessed with no significant differences among the three donors. At 4 h post-irradiation, let-7g, miR-21, miR-145, miR-663, miR-6727-5p, and miR-7641 were upregulated in a dose-dependent manner from 0 Gy to 6 Gy or up to 8 Gy, except for miR-7641, with an expression valley at 6 Gy (Figure 7). The expression of miR-34a and miR-3197 only increased after 4 Gy radiation, that of miR-1273g-3p increased at 8 Gy and 10 Gy radiation, and the others displayed no obvious changes (data not shown). At 24 h post-irradiation, the expression of miR-21, miR-6090, miR-6727-5p and miR-7641 were upregulated in a dose-dependent manner from 0 Gy to 4 Gy or up to 6 Gy; that of miR-1273g-3p and miR-1307-3p increased from 0 Gy to 8 Gy; that of miR-663 increased through 0 to 10 Gy with no difference among difference doses; and that of miR-3197 expression increased in 8 Gy and 10 Gy (Figure 8).

Base on the cumulative results, miR-145, miR-663, miR-1273g-3p, miR-miR-6090, miR-6727-5p, and miR-7641 were found to be dose-dependently upregulated by radiation in both AHH-1 cells and HPBLs at 4 h or 24 h post-irradiation. In addition, several miRNAs were upregulated by radiation at certain doses or in a certain dose range. These differentially expressed miRNAs signatures may serve as biomarkers or biodosimeter for the evaluation of the results of radiation exposure.

## 4. Discussion

Nuclear radiation accident usually breaks out unpredictably and can result in serious consequences to the public. Likewise, in deep space, the galactic cosmic rays (GCR) varies inversely with solar activity, the exposure to space radiation from solar storms is also unpredictable, which presents an acute risk to astronauts [25]. Obviously, it is an important to develop the accurate, simple, and quick response biomarkers or indicators for radiation emergency preparedness. Due to the relative stability at room temperature or after undergoing repeat freeze-thaw cycles and easily detection with the standard qRT-PCR, miRNAs seem to be ideal biomarkers with the assay characteristics of high throughput, rapid processing, high specificity, and sensitivity. Here, we screened the radiation-induced differential expression miRNAs through microarray analysis and further identified a panel of 15 miRNAs signature upregulated in response to radiation, among which 6 miRNAs were confirmed to shown increased expression after irradiation in a dose-dependent manner.

The major function of miRNAs is to epigenetically regulate the expression of target gene, through which it can function in a series processes of cellular responses to exogenous stresses. One mioRNA usually possesses multiples of target genes. We previously reported that increased miR-7-5p in the exosomes from the irradiated cells can induce the bystander autophagy though the targeting *EGFR* and *Bcl*2 in unirradiated cultured cells in vitro and in mice lung tissues in vivo [26,27]. The radiation-increased exosomal miR-1246 expression can induce bystander DNA damage in unirradiated cells by targeting the Lig4, which is a critical component in the non-homologous end joining pathway of DNA double-strand breaks repair [28]. Here the GO analyses of the top 6 upregulated miRNAs identified through microarray indicated that the top three clusters of MFs of the target genes are ubiquitin-protein transferase activity, DNA binding activity, and protein kinase activity. Notably, *DNAH3* and *GOLGB1* are target genes related to a maximum of four miRNAs. Dynein axonemal heavy chain 3 (*DNAH3*) is a member of dynein family that possess constituents of the microtubule-associated motor protein complex [29]. *GOLGB1* (giantin) is a widely expressed large coiled-coil protein and the largest Golgi matrix protein that functions in the cis-medial Golgi compartments. Localization and stability of dynein-2 at the Golgi complex could provide a mechanism to link axoneme initiation to membrane dynamics at the Golgi [30]. *GOLGB1* depletion can cause a moderate dispersion of Golgi membranes concomitant with the defect in ciliogenesis. *GOLGB1* was reported to function in ciliogenesis by controlling the localization of dynein-2 [30,31]. It seems both *GOLGB1* and *DNAH3* are involves in the reorganization and apparatus positioning of Golgi membranes and that there is no direct evidence or report linking *GOLGB1* and *DNAH3* belonging dynein family. Although there is no direct evidence or report linking GOLGB1 and DNAH3 with the cellular response to ionizing radiation, it has been reported that DNA damage, including radiation-induced DNA double-strand breaks (DSB), can trigger Golgi dramatic reorganization, resulting in its fragmentation/dispersal throughout the cytoplasm. In this process of Golgi response to DNA damage, DNA-PKcs, a critical component in the NHEJ pathway of DNA DSBs repair, phosphorylates Golgi phosphoprotein 3 (GOLPH3), which in turn increases the interaction between *GOLPH3* and *MYO18A*. Consequently, MYO18A/F-actin applies a tensile force to the Golgi [32]. This reorganization of Golgi apparatus was suggested to facilitate the cell survival after radiation exposure.

Our study that verified a panel of radiation-upregulated miRNAs, some of which are probably the first reported ones to be upregulated by ionizing radiation, including miR-1273g-3p, miR-1307-3p, miR-3197, miR-4324, miR-4638-5p, miR-5096, miR-6090, miR-6727-5p, and miR-7641. The miR-6090, miR-6727-5p, and miR-7641 were upregulated at 4 h or 24 h post-irradiation in dose-dependent manner in both AHH-1 and human peripheral blood lymphocytes irradiated ex vivo. In addition, the radiation-induced expression of some miRNAs recorded in our study have also been reported previously, including let-7e, let-7g, miR-21, miR-34a, miR-145, and miR-663 (Table S6), of which miR-145 and miR-663 were dose-dependently upregulated in both AHH-1 cells and HPBLs irradiated ex vivo. There are a large number of reports regarding the regulating roles of miRNAs on cellular response to irradiation. For example, Shi et al. reported radiation-induced miR-21 is continually elevated long-term in the brain [33] and radiation-induced miR-21 plays an important function in radiation-induced bystander effect [34,35], epithelium-to-mesenchymal transition (EMT) [14], DNA DSBs repair [36].

Multi-miRNA prognostic model has been established in several cancer types. A 3-miRNA signature exists as a novel potential prognostic biomarker in patients with clear cell renal cell carcinoma [37]. Not only for the prognostic purpose, a panel of miRNA signature in the plasma has been increasingly recognized as potential biomarker for diagnosis [38,39], treatment [40], and survival [41,42] of various tumors. In this study, in order to develop the new miRNA biomarkers and biodosimeter, the changes in the miRNA expression were detected at 4 h and 24 h time-points after irradiation, as they are more reasonable time frames to collect samples from early to late time range after radiation exposure. In the early stages of ionizing radiation, the increased expressions of 13 miRNAs was observed in AHH-1 cell; this increase in the expressions of miR-145, miR-3197, miR-4324, miR-6090 and miR-7641 was dose-dependent (Figure 3), and another eight miRNAs were upregulated by a certain dose(s) (Figure 4). At 24 h after irradiation, the expressions of miR-663, miR-1273g-3p, miR-3197, miR-4638-5p, miR-5090, miR-6090, miR-6727-5p, and miR-7641 were increased in a dose-dependent manner from 0–8 Gy or up to 10 Gy (Figure 5), while another seven miRNAs were upregulated at certain dose or dose range (Figure 6). In human peripheral blood samples irradiated ex vivo, let-7g, miR-21, miR-145, miR-663, miR-6727-5p, and miR-7641 were upregulated in a dose-dependent manner from 0 to 6 Gy or up to 8 Gy at 4 h after irradiation (Figure 7), while expression of miR-21, miR-6090, miR-6727-5p, and miR-7641 were upregulated in a dose-dependent manner from 0 to 4 Gy or up 6 Gy at 24 h after irradiation (Figure 8). Notably, the increased expressions of miR-145, miR-663, miR-1273g-3p, miR-miR-6090, miR-6727-5p, and miR-7641 were dose-dependent in both AHH-1 cells and the human peripheral blood cells. This panel of 6 miRNAs expression signature provided the prospect of developing early and rapid new biodosimeter of radiation exposure.

We noted some limitations in our study that should be mentioned. First, the expression levels of six miRNAs in HPBLs isolated from three healthy volunteers. More number of individuals should be tested, and the potential difference in age and sex should be further clarified. Second, we did not perform a standard curve for the quantitative estimation of the radiation doses about the six miRNAs signature; hence, further studies using larger sample sizes of HPBLs irradiated ex vivo or HPBLs from the patients before and after receiving radiotherapy would be helpful to further confirm the relationship between the radiation dose and alterations in the miRNA expression.

In conclusion, we identified a series of differentially expressed miRNAs induced by ionizing radiation, and thereby demonstrated that a new panel of miRNAs signature, including miR-145, miR-663, miR-1307-3p, miR-6090, miR-6727-5p, and miR-7541, which were upregulated in vitro and ex vivo after exposure to ionizing radiation. This six-miRNA signature possesses the potential of translational utility as a biomarker or biodosimeter to evaluate the radiation exposure and for rapid screening the individuals in the emergency management of radiation accidents.

## Figures and Tables

**Table 1:** The sorted top 6 of significantly changed miRNAs after 4 Gy of γ-ray irradiation *.

miRNA	Change Direction	Fold Change	Time Point	*p*-Value
hsa-miR-3197	Up	16.69	0.5 h	<0.001
	Up	5.23	2 h	<0.001
hsa-miR-1246	Up	1.03	0.5 h	<0.001
	Up	4.06	2 h	<0.001
hsa-miR-1307-3p	Up	2.39	0.5 h	<0.001
	UP	4.14	2 h	<0.001
hsa-miR-4267	Up	4.11	0.5 h	<0.001
	Down	1.69	2 h	<0.001
hsa-miR-5096	Up	14.92	0.5 h	<0.001
	UP	2.42	2 h	<0.001
hsa-miR-7641	UP	1.10	0.5 h	<0.001
	UP	3.45	2 h	<0.001

* Note: For each sample, miRNA expression was normalized to the U6 snRNA gene, and we calculated the expression levels relative to the control, 0 Gy group, *p* < 0.05.

## Supplementary Materials

The following data are available online at https://www.mdpi.com/2075-1729/10/12/361/s1. Figure S1: Heatmaps of differentially expressed miRNAs in HUVEC cells before and 0.5 h after 4 Gy of γ-ray. Fold changes of miRNAs are presented in the forms of natural logarithm, and bars in green refer to signals of microarrays of each miRNA in HUVEC cells. Figure S2: Heatmaps of differentially expressed miRNAs in HUVEC cells before and 2 h after 4 Gy of γ-ray. Fold changes of miRNAs are presented in the forms of natural logarithm, and bars in green refer to signals of microarrays of each miRNA in HUVEC cells. Figure S3: miRNA-gene network of significantly differentially expressed miRNAs and their predicted target genes in HUVEC cells before and after 4 Gy of γ-ray. A link was placed between a miRNA and a gene if the gene was predicted to be targeted by the miRNAs. Nodes in red refer to miRNAs while the blue refer to the genes. The size of nodes is proportional to their degrees. The 7 significantly differentially expressed miRNAs and genes targeted by > 3 miRNAs were marked. Table S1: The specific primer sequences for the PCR detection of miRNAs expression. Table S2: The fold change values and the *p* values of miRNA detection signal at 0.5 h after 4 Gy of γ-ray. Table S3: The fold change values and the *p* values of miRNA detection signal at 2 h after 4 Gy of γ-ray. Table S4: Enriched pathways of 396 predicted target genes related to >1 significantly differentially expressed miRNA. Table S5: The prediction of target genes of significantly differentially expressed miRNAs in HUVEC cells before and after with 4 Gy of γ-ray by DIANA TOOLS. Table S6: Radiation-upregulated miRNAs in the current study as well as in previous ones.

## References

[1] Takamura N., Orita M., Saenko V., Yamashita S., Nagataki S., Demidchik Y. (2016). Radiation and risk of thyroid cancer: Fukushima and Chernobyl. Lancet Diabetes Endocrinol..

[2] Mettler F.A., Gus’Kova A.K., Gusev I. (2007). Health effects in those with acute radiation sickness from the Chernobyl accident. Health Phys..

[3] Kamiya K., Ozasa K., Akiba S., Niwa O., Kodama K., Takamura N., Zaharieva E.K., Kimura Y., Wakeford R. (2015). Long-term effects of radiation exposure on health. Lancet.

[4] Chen Y., Zhou P.-K., Zhang X.-Q., Wang Z.-D., Wang Y., Darroudi F. (2014). Cytogenetic studies for a group of people living in Japan 1 year after the Fukushima nuclear accident. Radiat. Prot. Dosim..

[5] Chancellor J.C., Scott G.B.I., Sutton J.P. (2014). Space Radiation: The Number One Risk to Astronaut Health beyond Low Earth Orbit. Life.

[6] Obe G., Johannes I., Johannes C., Hallman K., Reitz G., Facius R. (1997). Chromosomal aberrations in blood lymphocytes of astronauts after long-term space flights. Int. J. Radiat. Biol..

[7] Evans H.J., Buckton K.E., Hamilton G.E., Carothers A. (1979). Radiation-induced chromosome aberrations in nuclear-dockyard workers. Nat. Cell Biol..

[8] Bianchi M., Bianchi N., Brewen J., Buckton K., Fabry L., Fischer P., Gooch P., Kučerová M., Léonard A., Mukherjee R. (1982). Evaluation of radiation-induced chromosomal aberrations in human peripheral blood lymphocytes in vitro results of an IAEA-coordinated programme. Mutat. Res. Mol. Mech. Mutagen..

[9] Andrei A., Wilkins R.C. (2009). The response ofgamma-H2AX in human lymphocytes and lymphocytes subsets measured in whole blood cultures. Int. J. Radiat. Biol..

[10] Huang R., Zhou P.-K. (2019). Double-edged effects of noncoding RNAs in responses to environmental genotoxic insults: Perspectives with regards to molecule-ecology network. Environ. Pollut..

[11] Wei W., He J., Wang J., Ding N., Wang B., Lin S., Zhang X., Hua J., Li H., Hu B. (2017). Serum microRNAs as Early Indicators for Estimation of Exposure Degree in Response to Ionizing Irradiation. Radiat. Res..

[12] Wei W., Wang J., He J., Xie X.D. (2018). Serum microRNA as noninvasive indicator for space radiation. Acta Astronaut..

[13] Huang R., Yu T., Li Y., Huang R. (2018). Upregulated has-miR-4516 as a potential biomarker for early diagnosis of dust-induced pulmonary fibrosis in patients with pneumoconiosis. Toxicol. Res..

[14] Liu Z., Liang X., Li X., Liu X., Zhu M., Gu Y., Zhou P. (2019). MiRNA-21 functions in ionizing radiation-induced epithelium-to-mesenchymal transition (EMT) by downregulating PTEN. Toxicol. Res..

[15] Martinez M., Rossetto I.M.U., Arantes R.M.S., Lizarte F.S.N., Tirapelli L.F., Tirapelli D.P.C., Chuffa L.G.A., Martinez F.E. (2019). Serum miRNAs are differentially altered by ethanol and caffeine consumption in rats. Toxicol. Res..

[16] Małachowska B., Tomasik B., Stawiski K., Kulkarni S., Guha C., Chowdhury D., Fendler W. (2020). Circulating microRNAs as Biomarkers of Radiation Exposure: A Systematic Review and Meta-Analysis. Int. J. Radiat. Oncol..

[17] Ménard C., Johann D., Lowenthal M., Muanza T., Sproull M., Ross S., Gulley J., Petricoin E., Coleman C.N., Whiteley G. (2006). Discovering Clinical Biomarkers of Ionizing Radiation Exposure with Serum Proteomic Analysis. Cancer Res..

[18] Sproull M., Kramp T., Tandle A., Shankavaram U., Camphausen K. (2015). Serum Amyloid A as a Biomarker for Radiation Exposure. Radiat. Res..

[19] Neto J.D.O.V., Da Silva C.A., Meneses G.C., Pinto D.V., Brito L.C., Fonseca S.G.D.C., Alves R.D.S., Martins A.M.C., Assumpção C.D.O., Daher E.D.F. (2020). Novel renal biomarkers show that creatine supplementation is safe: A double-blind, placebo-controlled randomized clinical trial. Toxicol. Res..

[20] Qiu X., Miao Y., Geng X., Zhou X., Li B. (2020). Evaluation of biomarkers for in vitro prediction of drug-induced nephrotoxicity in RPTEC/TERT1 cells. Toxicol. Res..

[21] Wei W., Bai H., Feng X., Hua J., Long K., He J., Zhang Y., Ding N., Wang J., Zhou H. (2020). Serum Proteins as New Biomarkers for Whole-Body Exposure to High- and Low-LET Ionizing Radiation. Dose-Response.

[22] Chipman L.B., Pasquinelli A.E. (2019). miRNA Targeting: Growing beyond the Seed. Trends Genet..

[23] Wang J., Liu S., Shi J., Li J., Wang S., Liu H., Zhao S., Duan K., Pan X., Yi Z. (2019). The Role of miRNA in the Diagnosis, Prognosis, and Treatment of Osteosarcoma. Cancer Biother. Radiopharm..

[24] Pasi F., Corbella F., Baio A., Capelli E., De Silvestri A., Tinelli C., Nano R. (2020). Radiation-induced circulating miRNA expression in blood of head and neck cancer patients. Radiat. Environ. Biophys..

[25] Norbury J.W., Slaba T.C., Aghara S., Badavi F.F., Blattnig S.R., Clowdsley M.S., Heilbronn L.H., Lee K., Maung K.M., Mertens C.J. (2019). Advances in space radiation physics and transport at NASA. Life Sci. Space Res..

[26] Song M., Wang Y., Shang Z.-F., Liu X.-D., Xie D.-F., Wang Q., Guan H., Zhou P.-K. (2016). Bystander autophagy mediated by radiation-induced exosomal miR-7-5p in non-targeted human bronchial epithelial cells. Sci. Rep..

[27] Cai S., Shi G.-S., Cheng H.-Y., Zeng Y.-N., Li G., Zhang M., Song M., Zhou P.-K., Tian Y., Cui F.-M. (2017). Exosomal miR-7 Mediates Bystander Autophagy in Lung after Focal Brain Irradiation in Mice. Int. J. Biol. Sci..

[28] Mo L.-J., Song M., Huang Q.-H., Guan H., Liu X.-D., Xie D.-F., Huang B., Huang R.-X., Zhou P.-K. (2018). Exosome-packaged miR-1246 contributes to bystander DNA damage by targeting LIG4. Br. J. Cancer.

[29] Hamdi Y., Boujemaa M., Ben Rekaya M., Ben Hamda C., Mighri N., El Benna H., Mejri N., Labidi S., Daoud N., The PEC Consortium (2018). Family specific genetic predisposition to breast cancer: Results from Tunisian whole exome sequenced breast cancer cases. J. Transl. Med..

[30] Asante D., MacCarthy-Morrogh L.J., Townley A.K., Weiss M.A., Katayama K., Palmer K.J., Suzuki H., Westlake C.J., Stephens D.J. (2013). A role for the Golgi matrix protein giantin in ciliogenesis through control of the localization of dynein-2. J. Cell Sci..

[31] Bergen D.J., Stevenson N.L., Skinner R.E.H., Stephens D.J., Hammond C.L. (2017). The Golgi matrix protein giantin is required for normal cilia function in zebrafish. Biol. Open.

[32] Farber-Katz S.E., Dippold H.C., Buschman M.D., Peterman M.C., Xing M., Noakes C.J., Tat J., Ng M.M., Rahajeng J., Cowan D.M. (2014). DNA Damage Triggers Golgi Dispersal via DNA-PK and GOLPH3. Cell.

[33] Shi Y., Zhang X., Tang X., Wang P., Wang H., Wang Y. (2011). MiR-21 is continually elevated long-term in the brain after exposure to ionizing radiation. Radiat. Res..

[34] Xu S., Ding N., Pei H., Hu W., Wei W., Zhang X., Zhou G., Wang J. (2014). MiR-21 is involved in radiation-induced bystander effects. RNA Biol..

[35] Yin X., Tian W., Wang L., Wang J., Zhang S., Cao J., Yang H. (2015). Radiation quality-dependence of bystander effect in unirradiated fibroblasts is associated with TGF-β1-Smad2 pathway and miR-21 in irradiated keratinocytes. Sci. Rep..

[36] Hu B., Wang X., Hu S., Ying X., Wang P., Zhang X., Wang J., Wang H., Wang Y. (2017). miR-21-mediated Radioresistance Occurs via Promoting Repair of DNA Double Strand Breaks. J. Biol. Chem..

[37] Luo Y., Chen L., Wang G., Xiao Y., Ju L., Wang X. (2019). Identification of a three-miRNA signature as a novel potential prognostic biomarker in patients with clear cell renal cell carcinoma. J. Cell. Biochem..

[38] Zhang H., Zhu M., Shan X., Zhou X., Wang T., Zhang J., Tao J., Cheng W., Chen G., Li J. (2018). A panel of seven-miRNA signature in plasma as potential biomarker for colorectal cancer diagnosis. Gene.

[39] Huang C., Wang Q., Ma S., Sun Y., Vadamootoo A.S., Jin C. (2019). A four serum-miRNA panel serves as a potential diagnostic biomarker of osteosarcoma. Int. J. Clin. Oncol..

[40] Li Z., Ye L., Wang L., Quan R., Zhou Y., Li X. (2020). Identification of miRNA signatures in serum exosomes as a potential biomarker after radiotherapy treatment in glioma patients. Ann. Diagn. Pathol..

[41] Zhou H., Tang K., Xiao H., Zeng J., Guan W., Guo X., Xu H., Ye Z. (2015). A panel of eight-miRNA signature as a potential biomarker for predicting survival in bladder cancer. J. Exp. Clin. Cancer Res..

[42] Wu Y.-S., Lin H., Chen D., Yi Z., Zeng B., Jiang Y., Ren G. (2019). A four-miRNA signature as a novel biomarker for predicting survival in endometrial cancer. Gene.

