# Supplementary material for: A Biomarker Panel of Radiation-Upregulated miRNA as Signature for Ionizing Radiation Exposure
Source: Life (Basel). 2020 Dec 18;10(12):361. doi: 10.3390/life10120361 (PMC7766228; doi:10.3390/life10120361)
Supplement: Supplementary file 1 [file life-10-00361-s001.zip › Legends of Supplementary Materials.pdf]

Legends of Supplementary Materials

Figure S1: Heatmaps of differentially expressed miRNAs in HUVEC cells before and 0.5-h after irradiation with 4-Gy γ-rays. Fold changes of miRNAs are presented in the forms of natural logarithm, and bars in green refer to signals of microarrays of each miRNA in the HUVEC cells.

Figure S2: Heatmaps of differentially expressed miRNAs in HUVEC cells before and 2h after irradiation with 4-Gy γ-rays. Fold changes of miRNAs are presented in the forms of natural logarithm, and bars in green refer to signals of microarrays of each miRNA in the HUVEC cells.

Figure S3: miRNA-gene network of significantly differentially expressed miRNAs and their predicted target genes in HUVEC cells before and after irradiation with 4-Gy γ-rays. A link was placed between an miRNA and a gene if the gene was predicted to be targeted by the miRNAs. Nodes in red refer to miRNAs while those in blue refer to the genes. The size of nodes is proportional to their degrees. The 7 significantly differentially expressed miRNAs and genes targeted by >3 miRNAs were marked.

Table S1: The specific primer sequences for the PCR detection of miRNAs expression.

Table S2: The fold change values and the p values of miRNA detection signal at 0.5 h after 4 Gy of γ-ray.

Table S3: The fold change values and the p values of miRNA detection signal at 2 h after 4 Gy of γ-ray.

Table S4: Enriched pathways of 396 predicted target genes related to > 1 significantly differentially expressed miRNA.

Table S5: The prediction of target genes of significantly differentially expressed miRNAs in HUVEC cells before and after with 4 Gy of γ-ray by DIANA TOOLS.

Table S6: Radiation-upregulated miRNAs in the current study as well as in previous ones.
