# Supplementary material for: A Biomarker Panel of Radiation-Upregulated miRNA as Signature for Ionizing Radiation Exposure
Source: Life (Basel). 2020 Dec 18;10(12):361. doi: 10.3390/life10120361 (PMC7766228; doi:10.3390/life10120361)
Supplement: Supplementary file 1 [file life-10-00361-s001.zip › Table S1.pdf]

Table S1. The specific primer sequences for the PCR detection of miRNAs expression

miRNA	PCR Primer sequence
Has-let-7e-Forword primer	TGCCGCTGAGGTAGGAGG
Has-let-7e-Reverse primer	TGGAGCCTGGGACGAGA
Has-let-7g-Forword primer	GCCGCTGAGGTAGTAGTTTGTA
Has-let-7g-Reverse primer	TGGAGCCTGGGACGAGA
Has-miR-21-Forword primer	TCGCCCGTAGCTTATCAGACT
Has-miR-21-Reverse primer	CAGAGCAGGGTCCGAGGTA
Has-miR-34a-Forword primer	AGCCGCTGGCAGTGTCTTA
Has-miR-34a-Reverse primer	CAGAGCAGGGTCCGAGGTA
Has-miR-145-Forword primer	TGCTCTGGATTCCTGGAAAT
Has-miR-145-Reverse primer	TATGGTTGTTCTGCTCTCTGTCTC
Has-miR-663-Forword primer	GACTCAATAGGCGGGGCG
Has-miR-663-Reverse primer	TATGGTTGTTCACGACTCCTTCAC
Has-miR-1273g-3p-Forword primer	AGACCACACCACTGCACTCC
Has-miR-1273g-3p-Reverse primer	CAGAGCAGGGTCCGAGGTA
Has-miR-1307-3p-Forword primer	GGACTCGGCGTGGCGT
Has-miR-1307-3p-Reverse primer	TATGGTTGTTCACGACTCCTTCAC
Has-miR-3197-Forword primer	TCCGAGGGAGGCGCAGGC
Has-miR-3197-Reverse primer	TGGAGCCTGGGACGAGA
Has-miR-4324-Forword primer	CTTCCCTTCCCTGAGACCCT
Has-miR-4324-Reverse primer	TATGGTTGTTCACGACTCCTTCAC
Has-miR-4638-5p-Forword primer	GGGTTTCCTGGACACCGCTC
Has-miR-4638-5p-Reverse primer	TATGGTTGTTCACGACTCCTTCAC
Has-miR-5096-Forword primer	GGTTCCCAAGTTTCACCATGTT
Has-miR-5096-Reverse primer	TATGGTTGTTCACGACTCCTTCAC
Has-miR-6090-Forword primer	TTCCTTTGGGGAGCGAGG
Has-miR-6090-Reverse primer	TATGGTTGTTCACGACTCCTTCAC
Has-miR-6727-5p-Forword primer	CTCGGGGCAGGCGG
Has-miR-6727-5p-Reverse primer	TATGGTTGTTCACGACTCCTTCAC
Has-miR-7641-Forword primer	CGCTGGGTTGATCTCGGA
Has-miR-7641-Reverse primer	CAGTTGGTCAGCAGTTCTTGGTAT
U6 snRNA-Forword primer	CGCTTCGGCAGCACATATAC
U6 snRNA-Revere primer	TTCACGAATTTGCGTGTCATA
