# Supplementary material for: A Biomarker Panel of Radiation-Upregulated miRNA as Signature for Ionizing Radiation Exposure
Source: Life (Basel). 2020 Dec 18;10(12):361. doi: 10.3390/life10120361 (PMC7766228; doi:10.3390/life10120361)
Supplement: Supplementary file 1 [file life-10-00361-s001.zip › Table S6.pdf]

Table S6 Radiation-upregulated miRNAs in current study as well as previous reports

miRNA	Cell line	Dose(Gy)	Collection time	Method	Expression	Ref
let-7e	TK6	2Gy	0.5,4,8,12,24h	qRT-PCR	↑ at 4h	[1]
	AG1522	10Gy	1h	microarrays	↑ at 1h	[2]
	M059K	3Gy	4, 8, 12, 24h	qRT-PCR	↑ at 4,8,12,24h	[3]
let-7g	HDMEC	2Gy	6h	microarrays	↑ at 6h	[4]
	U87MG	18.8Gy	4h	microarrays	↑ at 4h	[5]
	blood	1.25 Gy	4h	qRT-PCR	↑ at 4h	[6]
miR-21	HaCaT	1	1h,3h	qRT-PCR	↑ at 3h	[7]
	TE-1	2,4,6,8	24h	qRT-PCR	↑ at 24h	[8]
	A549	6, 8	24h	qRT-PCR	↑ at 24h	[9]
	U251	10	3,6,12,24h	qRT-PCR	↑ at 3,6,12h	[10]
miR-34a	A549	40	24h	microarrays	↑ at 24h	[11]
	IM9	10	24h	microarrays	↑ at 24h	[12]
	serum	50	5 weeks	qRT-PCR	↑ at 5 weeks	[13]
miR-663	AG01522	10	1h	microarrays	↑ at 1h	[2]
	IM9	1, 10	24h	microarrays	↑ at 24h	[14]
	HFL-1	0.5,1.0	24h	qRT-PCR	↑ at 24h	[15]
miR-145	TK6	2	0.5,4,8,12,24h	qRT-PCR	↑ at 4h, 24h	[1]
	HeLa	4,6,8	24h	qRT-PCR	↑ at 24h	[16]
	blood	1.25	4h	qRT-PCR	↑ at 4h	[6]

1. Chaudhry, M.A.; Omaruddin, R.A. Differential regulation of microRNA expression in irradiated and bystander cells. *Mol Biol (Mosk)* **2012**, *46*, 634-643.
2. Simone, N.L.; Soule, B.P.; Ly, D.; Saleh, A.D.; Savage, J.E.; Degraff, W.; Cook, J.; Harris, C.C.; Gius, D.; Mitchell, J.B. Ionizing radiation-induced oxidative stress alters miRNA expression. *PLoS One* **2009**, *4*, e6377, doi:10.1371/journal.pone.0006377.
3. Chaudhry, M.A.; Sachdeva, H.; Omaruddin, R.A. Radiation-induced micro-RNA modulation in glioblastoma cells differing in DNA-repair pathways. *DNA Cell Biol* **2010**, *29*, 553-561, doi:10.1089/dna.2009.0978.
4. Wagner-Ecker, M.; Schwager, C.; Wirkner, U.; Abdollahi, A.; Huber, P.E. MicroRNA expression

after ionizing radiation in human endothelial cells. *Radiat Oncol* **2010**, *5*, 25, doi:10.1186/1748-717X-5-25.
5. Chen, G.; Zhu, W.; Shi, D.; Lv, L.; Zhang, C.; Liu, P.; Hu, W. MicroRNA-181a sensitizes human malignant glioma U87MG cells to radiation by targeting Bcl-2. *Oncol Rep* **2010**, *23*, 997-1003, doi:10.3892/or_00000725.
6. Templin, T.; Paul, S.; Amundson, S.A.; Young, E.F.; Barker, C.A.; Wolden, S.L.; Smilenov, L.B. Radiation-induced micro-RNA expression changes in peripheral blood cells of radiotherapy patients. *Int J Radiat Oncol Biol Phys* **2011**, *80*, 549-557, doi:10.1016/j.ijrobp.2010.12.061.
7. Yin, X.; Tian, W.; Wang, L.; Wang, J.; Zhang, S.; Cao, J.; Yang, H. Radiation quality-dependence of bystander effect in unirradiated fibroblasts is associated with TGF-beta1-Smad2 pathway and miR-21 in irradiated keratinocytes. *Sci Rep* **2015**, *5*, 11373, doi:10.1038/srep11373.
8. Lin, J.; Liu, Z.; Liao, S.; Li, E.; Wu, X.; Zeng, W. Elevation of long non-coding RNA GAS5 and knockdown of microRNA-21 up-regulate RECK expression to enhance esophageal squamous cell carcinoma cell radio-sensitivity after radiotherapy. *Genomics* **2020**, *112*, 2173-2185, doi:10.1016/j.ygeno.2019.12.013.
9. Song, Y.; Zuo, Y.; Qian, X.L.; Chen, Z.P.; Wang, S.K.; Song, L.; Peng, L.P. Inhibition of MicroRNA-21-5p Promotes the Radiation Sensitivity of Non-Small Cell Lung Cancer Through HMSH2. *Cell Physiol Biochem* **2017**, *43*, 1258-1272, doi:10.1159/000481839.
10. Li, Y.; Zhao, S.; Zhen, Y.; Li, Q.; Teng, L.; Asai, A.; Kawamoto, K. A miR-21 inhibitor enhances apoptosis and reduces G(2)-M accumulation induced by ionizing radiation in human glioblastoma U251 cells. *Brain Tumor Pathol* **2011**, *28*, 209-214, doi:10.1007/s10014-011-0037-1.
11. Shin, S.; Cha, H.J.; Lee, E.M.; Lee, S.J.; Seo, S.K.; Jin, H.O.; Park, I.C.; Jin, Y.W.; An, S. Alteration of miRNA profiles by ionizing radiation in A549 human non-small cell lung cancer cells. *Int J Oncol* **2009**, *35*, 81-86.
12. An. Identification of ionizing radiation-responsive microRNAs in the IM9 human B lymphoblastic cell line. *International Journal of Oncology* **2009**, *34*, 1661---1668.
13. Halimi, M.; Shahabi, A.; Moslemi, D.; Parsian, H.; Asghari, S.M.; Sariri, R.; Yeganeh, F.; Zabihi, E. Human serum miR-34a as an indicator of exposure to ionizing radiation. *Radiat Environ Biophys* **2016**, *55*, 423-429, doi:10.1007/s00411-016-0661-6.
14. Cha, H.J.; Shin, S.; Yoo, H.; Lee, E.M.; Bae, S.; Yang, K.H.; Lee, S.J.; Park, I.C.; Jin, Y.W.; An, S. Identification of ionizing radiation-responsive microRNAs in the IM9 human B lymphoblastic cell line. *Int J Oncol* **2009**, *34*, 1661-1668, doi:10.3892/ijo_00000297.
15. Chauhan, V.; Howland, M.; Wilkins, R. Effects of alpha-Particle Radiation on MicroRNA Responses in Human Cell-Lines. *Open Biochem J* **2012**, *6*, 16-22, doi:10.2174/1874091X01206010016.
16. Ye, C.; Sun, N.X.; Ma, Y.; Zhao, Q.; Zhang, Q.; Xu, C.; Wang, S.B.; Sun, S.H.; Wang, F.; Li, W. MicroRNA-145 contributes to enhancing radiosensitivity of cervical cancer cells. *FEBS Lett* **2015**, *589*, 702-709, doi:10.1016/j.febslet.2015.01.037.
