# Supplementary figures and images for: A Biomarker Panel of Radiation-Upregulated miRNA as Signature for Ionizing Radiation Exposure
Source: Life (Basel). 2020 Dec 18;10(12):361. doi: 10.3390/life10120361 (PMC7766228; doi:10.3390/life10120361)

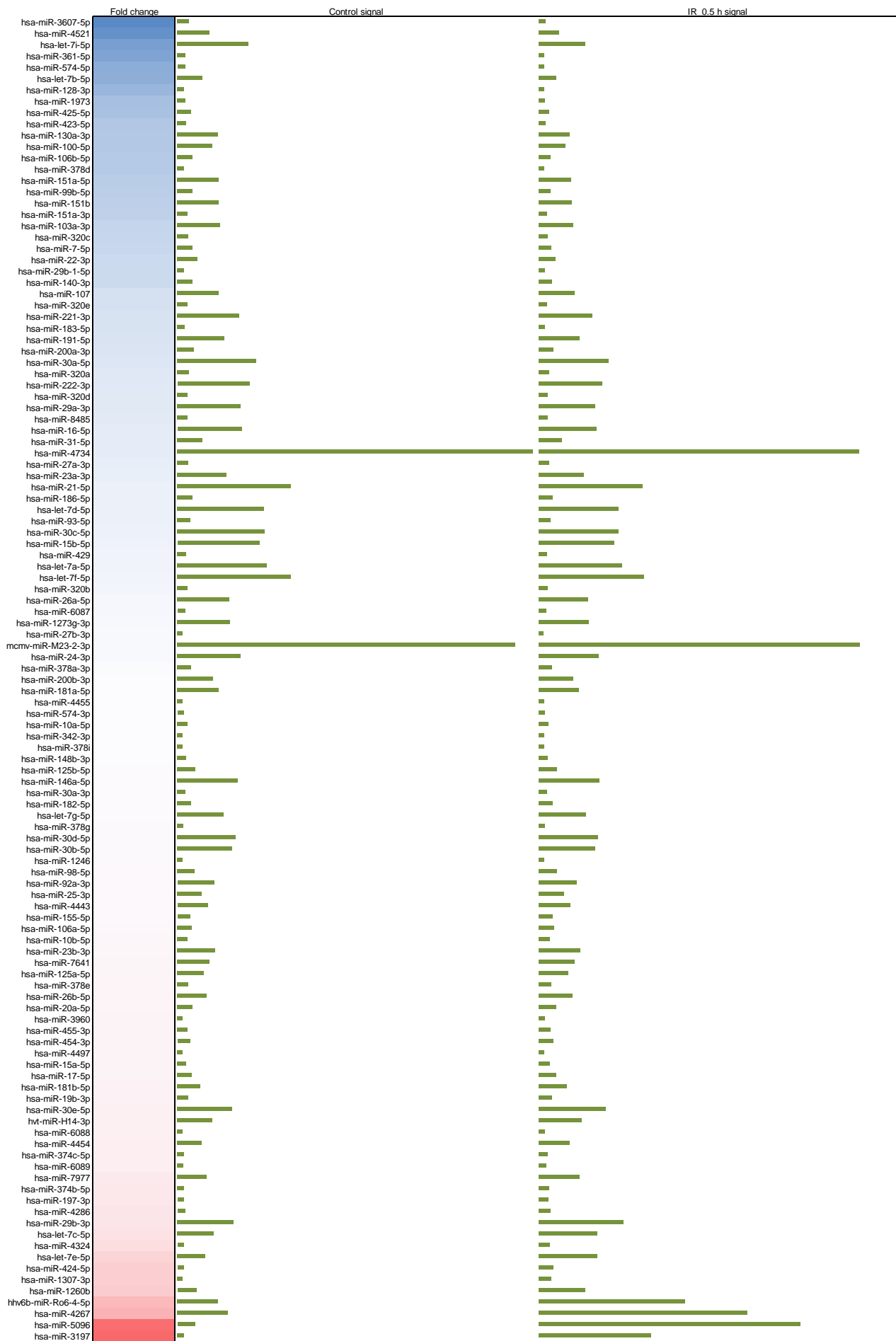

Supplement: Supplementary file 1 [file life-10-00361-s001.zip › Figure S1.pdf]

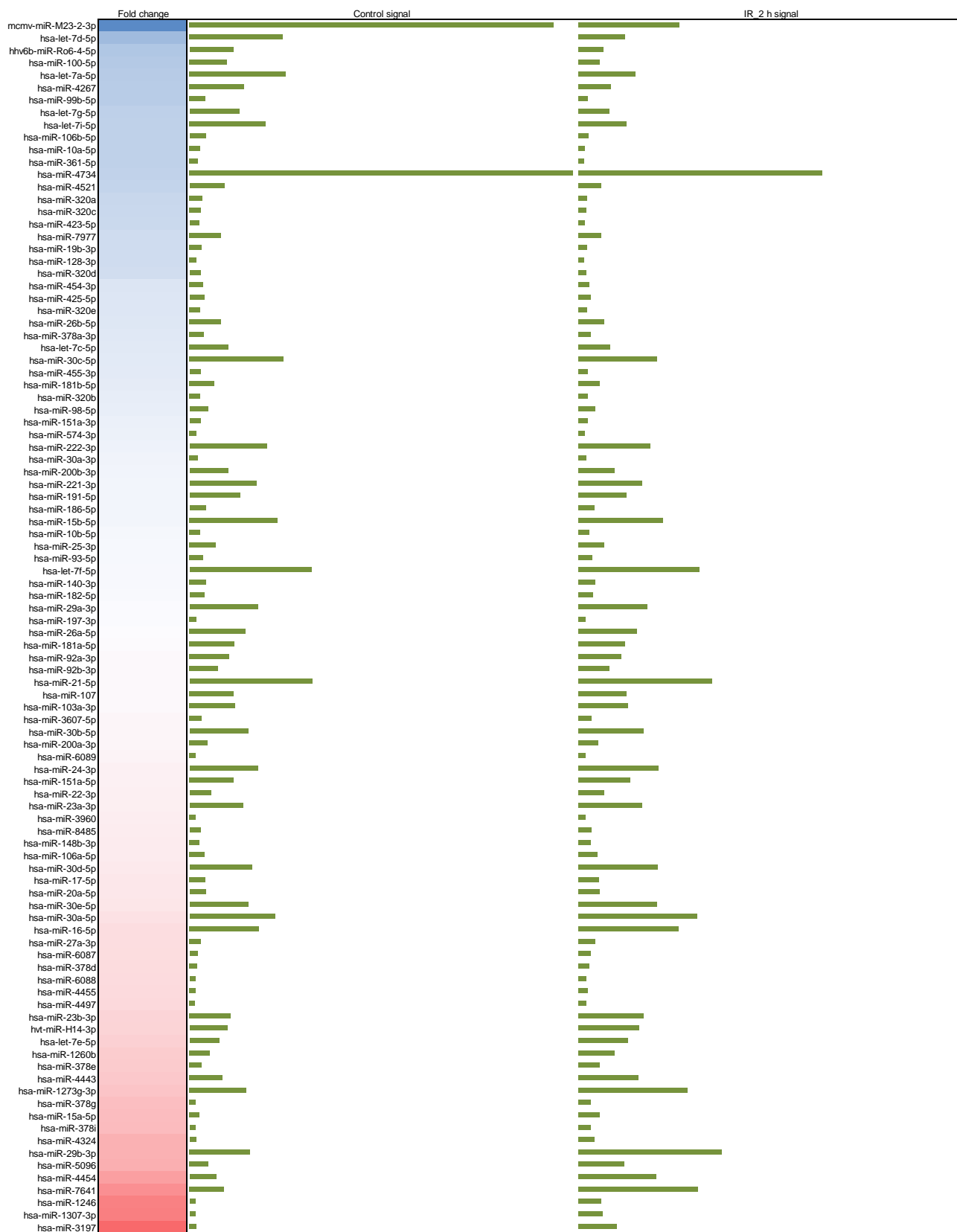

Supplement: Supplementary file 1 [file life-10-00361-s001.zip › Figure S2.pdf]

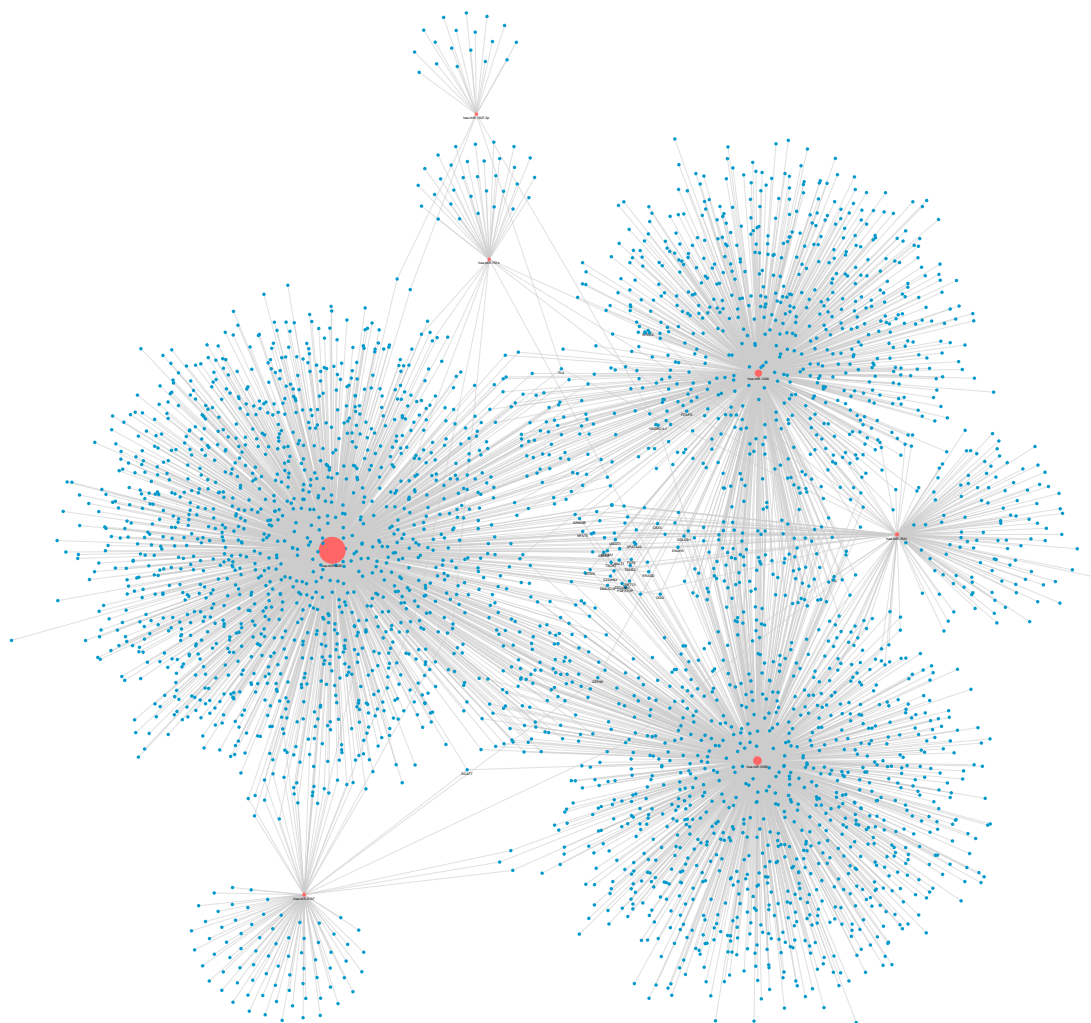

Supplement: Supplementary file 1 [file life-10-00361-s001.zip › Figure S3.pdf]
